# Preferential HLA-B27 Allorecognition Displayed by Multiple Cross-Reactive Antiviral CD8^+^ T Cell Receptors

**DOI:** 10.3389/fimmu.2020.00248

**Published:** 2020-02-19

**Authors:** Louise C. Rowntree, Heleen van den Heuvel, Jessica Sun, Lloyd J. D'Orsogna, Thi H. O. Nguyen, Frans H. J. Claas, Jamie Rossjohn, Tom C. Kotsimbos, Anthony W. Purcell, Nicole A. Mifsud

**Affiliations:** ^1^Respiratory Medicine Laboratory, Department of Medicine, Central Clinical School, Monash University, Melbourne, VIC, Australia; ^2^Department of Allergy, Immunology, and Respiratory Medicine, The Alfred Hospital, Melbourne, VIC, Australia; ^3^Infection and Immunity Program, Department of Biochemistry and Molecular Biology, Biomedicine Discovery Institute, Monash University, Clayton, VIC, Australia; ^4^Department of Immunohaematology and Blood Transfusion, Leiden University Medical Center, Leiden, Netherlands; ^5^Department of Clinical Immunology and Pathwest, Fiona Stanley Hospital, Perth, WA, Australia; ^6^School of Medicine, University of Western Australia, Perth, WA, Australia; ^7^Department of Microbiology and Immunology, Peter Doherty Institute for Infection and Immunity, The University of Melbourne, Parkville, VIC, Australia; ^8^Australian Research Council Centre of Excellence for Advanced Molecular Imaging, Monash University, Clayton, VIC, Australia; ^9^Institute of Infection and Immunity, Cardiff University School of Medicine, Heath Park, Cardiff, United Kingdom

**Keywords:** cross-reactivity, CMV, EBV, HIV-1, HLA, T cells, TCR

## Abstract

T cells provide essential immunosurveillance to combat and eliminate infection from pathogens, yet these cells can also induce unwanted immune responses via T cell receptor (TCR) cross-reactivity, also known as heterologous immunity. Indeed, pathogen-induced TCR cross-reactivity has shown to be a common, robust, and functionally potent mechanism that can trigger a spectrum of human immunopathologies associated with either transplant rejection, drug allergy, and autoimmunity. Here, we report that several virus-specific CD8^+^ T cells directed against peptides derived from chronic viruses (EBV, CMV, and HIV-1) presented by high frequency HLA-A and -B allomorphs differentially cross-react toward HLA-B27 allotypes in a highly focused and hierarchical manner. Given the commonality of cross-reactive T cells and their potential contribution to adverse outcomes in allogeneic transplants, our study demonstrates that multiple antiviral T cells recognizing the same HLA allomorph could pose an extra layer of complexity for organ matching.

## Introduction

A hallmark of human antiviral T cells is their ability to recognize viral peptide antigen bound to a self-human leukocyte antigen (HLA) on the surface of infected cells. Whilst this recognition often displays exquisite specificity, it is not uncommon for some of these T cells to cross-react with closely related peptide-HLA (pHLA) complexes, such as a peptide from a different viral strain (1). Given that T cells are inherently cross-reactive, by nature of thymic selection (i.e., recognition of self) and their interaction with foreign antigen in the periphery, cross-strain reactivity is a beneficial property affording protection to mutant viral strains and preventing immune escape. More remarkably, some T cells are also capable of recognizing apparently distinct pHLA including non-self or allogeneic pHLA (2–4), self-pHLA that have undergone some form of perturbation resulting in an altered self-peptide repertoire (5), and self-pHLA expressed in different tissues (6). These forms of heterologous immunity, otherwise known as T cell cross-reactivity, are not beneficial to the host and can lead to transplant rejection, drug hypersensitivity and autoimmunity, respectively. Moreover, these potentially hazardous T cell responses are the price paid to maintain immune potential to combat the vast array of pathogenic challenges during a lifetime. Hence, cross-reactivity is an intrinsic feature of T cells, necessitated by the limited availability of unique human T cell receptor (TCR) clonotypes (<10^8^ distinct TCRs) to maintain immunity against tremendous pathogenic diversity (>10^15^ pHLA combinations) (7).

Childhood exposure to common viruses results in the induction of a robust immune response that controls the infection and generates long lasting immune memory. A small proportion of some viruses (e.g., herpesviruses including Epstein-Barr virus [EBV] and cytomegalovirus [CMV]) are able to evade the immune response by entering into a latent state inside the host cells. In fact, for these common herpesviruses up to 90% of individuals maintain viral latency by adulthood (8). The persistence of a memory pool of T cells against the virus generally controls outbreaks of viral reactivation. Recurrent reactivation episodes maintain these memory T cells at high frequency, facilitating rapid deployment and activation. Virally triggered cross-reactive T cells have predominantly been explored in infections where there is a high likelihood of their relevance after transplantation. This is particularly so for EBV and CMV, which establish latency in the host following naturally acquired or vaccine-induced immunity. These viruses have been directly implicated as risk factors associated with allograft rejection and graft vs. host disease (8), with studies demonstrating that high frequencies of herpesvirus-derived cross-reactive T cells (up to 85%) or clones (up to 45%) co-recognize alternate HLA allotypes (3, 9–11). Whilst there is a high likelihood that cross-reactive T cells are involved in clinical rejection (11–13), this has yet to be formally proven.

Allo-HLA cross-reactivity by antiviral T cells has been reported across a variety of HLA class I (A and B loci) and II (DRB1 locus) restricted targets [reviewed in (14)]. In some instances, antiviral T cells derived from either the same or heterologous viruses are capable of recognizing an identical HLA allomorph. For instance, HLA-B^*^44:02 is cross-recognized by B^*^08:01-restricted LC13 cytotoxic T lymphocytes (CTL; EBV EBNA3A_325−333_), B^*^35:08-restricted SB27 CTL (EBV BZLF1_52−64_) and A^*^02:01-restricted 5101.1999.23 CTL (herpes simplex virus-2 VP13/14_289−298_) (15–19). Given the commonality of cross-reactive T cells and their potential to contribute to adverse immune responses in allogeneic transplants we wanted to determine whether multiple antiviral CTLs recognizing the same HLA allomorphs would contribute an extra layer of complexity for organ matching. This study examines the extent of T cell cross-reactivity generated by three heterologous viruses (i.e., EBV, CMV, and human immunodeficiency virus-1 [HIV-1]) toward different HLA-B27 allotypes, which may have clinical implications for transplantation.

## Materials and Methods

### Study Participants and Peripheral Blood Mononuclear Cells Isolation

Participant HLA typing is shown in Supplementary Table 1. Peripheral blood mononuclear cells (PBMC) were isolated by standard Ficoll-Paque (GE Healthcare, Uppsala, Sweden) density gradient centrifugation and cryopreserved at −196°C until required.

### Virus-Specific CD8^+^ T Cell Lines or Clones

EBV, CMV, or influenza A (IAV)-specific CD8^+^ T cell lines were generated from chronically-infected individuals following *in vitro* expansion of PBMC stimulated with gamma-irradiated peptide-pulsed autologous cells (1 μM peptide, 3,000 Rads) at a 2:1 ratio in RF10 [composed of RPMI 1640 (Life Technologies, Grand Island, NY) supplemented with 2 mM MEM non-essential amino acid solution (Life Technologies), 100 mM HEPES (Life Techologies), 2 mM L-glutamine (Life Technologies), penicillin/streptomycin (Life Technologies), 50 mM 2-mercaptoethanol (Sigma-Aldrich, St. Louis, MO), 10% heat-inactivated FCS (Sigma-Aldrich)] supplemented with 20 U/mL IL-2 (PeproTech, Rocky Hill, NJ) for 13 days at 37°C, 5% CO_2_ as previously described (4, 11). Peptides for CMV: HLA-A^*^02:01-restricted pp65-derived NLVPMVATV (A2_NLV_) epitope, EBV: HLA-B^*^07:02-restricted EBNA-3A-derived RPPIFIRRL (B7_RPP_) epitope and IAV: HLA-A^*^02:01-restricted matrix protein-derived GILGFVFTL (A2_GIL_) epitope. Virus-specific CD8^+^ T cell clones from chronically-infected individuals were generated following single-cell sorting based on tetramer staining using the HLA-B^*^57:01-restricted TSTLQEQIGW (B57_TW10_) epitope derived from HIV-1 Gag protein for A16 and 457 (20) or EBV: B7_RPP_ epitope for HD9G6 (21), as previously described (2, 22, 23).

### Antigen-Presenting Cells and HLA Cell Surface Expression

C1R transfected cells expressing different HLA-I molecules (HLA-A^*^02:01, -B^*^07:02, -B^*^57:01, -B^*^27:01 to -B^*^27:10) were used as antigen-presenting cells (APCs), maintained in RF10 with selection antibiotics [Geneticin G418 (0.4–0.5 mg/ml; Roche Diagnostics, Mannheim, Germany) or hygromycin B (0.3 mg/ml; Life Technologies, Carlsbad, CA)] as required (4, 24). Increased HLA-I expression [compared to C1R Parental, which has low levels of HLA-A and HLA-B expression and normal HLA-C (25)] was confirmed via flow cytometry by indirect staining with appropriate antibodies; anti-human pan HLA-I (W6/32 hybridoma; for C1R.A^*^02:01, C1R.B^*^07:02, C1R.B^*^57:01 shown in Supplementary Figure 1A), anti-human HLA-B7/27 (ME1 hybridoma; for C1R.B^*^27:01 to C1R.B^*^27:10 shown in Supplementary Figure 1B) and a secondary goat anti-mouse IgG phycoerythrin (PE) (1:200 dilution; Southern Biotech, Birmingham, AL). All hybridomas were produced in-house. Stained cells were acquired on LSRII flow cytometer [Becton Dickinson (BD), San Jose, CA]. Flow cytometry data was analyzed using FlowJo software (TreeStar, Ashland, OR).

### Specificity and Functionality of CD8^+^ T Cell Lines or Clones

The specificity and activation of virus-specific CD8^+^ T cells were assessed by anti-CD8 and tetramer (A2_NLV_ or B7_RPP_) co-staining, followed by intracellular staining (ICS) for functional Th1 cytokine production using flow cytometry (11, 26). Briefly, 2 × 10^5^ day 13 T cells were stimulated with media (negative control), Dynabeads® Human T-Activator anti-CD3/CD28 (positive control; Life Technologies) or 1 × 10^5^ APC (±1 μM peptide) for a total of 6 h at 37°C, 5% CO_2_ with 10 μg/mL brefeldin A (Sigma-Aldrich) added for the last 4 h. T cells were phenotyped with anti-CD8 PerCP Cy5.5 or allophycocyanin (APC) (1:20 or 1:40 dilution, clone SK1, BD Biosciences, San Jose, CA), HLA-A2_NLV_ or HLA-B7_RPP_ tetramer (conjugated to either PE or APC) and LIVE/DEAD fixable aqua stain (1:750 dilution, Thermo Fisher Scientific, Waltham, MA). T cells were then fixed in 1% paraformaldehyde (ProSciTech, Kirwan, Queensland, Australia), permeabilized in 0.3% saponin (Sigma-Aldrich) containing anti-IFNγ PE-Cy7 (1:250 dilution, clone B27, BD Biosciences) and anti-TNFα V450 (1:400 dilution, clone Mab11, BD Biosciences), then acquired on LSRII flow cytometer. Flow cytometry data was analyzed using FlowJo software. The HIV-1 B57_TW10_ CD8^+^ T cell clones, A16 and 457, were assessed for functionality toward cognate peptide by (i) staining of cell surface anti-CD3 V450 and anti-CD137 APC (BD Biosciences) for 20 min and then analyzed on the FACSCanto II (BD) according to standard procedures, and (ii) functional cytotoxicity against single HLA expressing K562 cell line loaded with cognate peptide, using target cell 7-AAD uptake as readout, as previously published (27). This study shows the data for A16, with 457 being published elsewhere (20). Gating strategies are shown in Supplementary Figure 2. Tetramers were produced in-house by refolding soluble HLA α-heavy chain-BirA and β2-microglobulin with peptide to create monomers, which were then conjugated at a 4:1 molar ratio to streptavidin-PE or -APC (Life Technologies) (24).

### αβTCR Identification

Virus-specific CD8^+^ T cells lines were incubated with 1 μM peptide or relevant peptide-pulsed C1R transfected cells for 2 h before detection of cytokine secretion using an anti-IFNγ antibody (IFNγ Secretion Assay Detection Kit APC; Miltenyi Biotec, Auburn, CA) as previously described (28). CD8^+^ T cells were single-cell sorted directly into semi-skirted 96-well plates (Bio-Rad Laboratories Inc., USA) based on tetramer specificity and ± IFNγ production (FACSAria I, BD Biosciences operated by FlowCore, Monash University). Sorted plates were immediately stored at −80°C until required. TCR analysis of paired complementarity determining region (CDR)3α and β loops were carried out using multiplex nested RT-PCR and sequencing of α and β gene products as previously described (29). For virus-specific CD8^+^ T cell clones, αβTCR usage was determined by DNA Sanger sequencing using either TCR-specific PCR for HD9G6 (30) or next-generation sequencing using published primer sequences (31) for A16 and 457.

### TCR Expression in SKW3.hCD8αβ Cells

Full-length human TCRα and TCRβ cDNA was cloned into a self-cleaving 2A peptide-based pMIG vector as described previously (32). HEK293T packaging cells were incubated with 4 mg pEQ-pam3(-E) and 2 mg pVSV-G packaging vectors, in the presence of 4 mg pMIG vector each containing a specific TCR transgene using Lipofectamine 3000 (Life Technologies). HEK293T cell culture supernatant containing virus particles carrying the TCR transgene was then used to retrovirally transduce GFP-tagged SKW3.hCD8αβ cells or GFP-tagged SKW3.hCD8αβ.CD3 [for LTR5 TCR only (28)], which are negative for endogenous TCRαβ but contain CD3 and signaling components, as previously described (28). SKW3.hCD8αβ.TCR (hereafter referred to as SKW3) cell lines were maintained in RF10. Routine monitoring of TCR cell surface expression on SKW3 transduced cells was performed using anti-CD3 PE-Cy7 (1:500 dilution, clone SK7, BD Biosciences), anti-CD8 PerCP Cy5.5 (1:20 dilution, clone SK1, BD Biosciences) and GFP. Gating strategy shown in Supplementary Figure 3. Activation of SKW3.TCRs were assessed via cell surface staining with anti-CD3 PE-Cy7 (1:500 dilution, clone SK7, BD Biosciences), anti-CD8 PerCP Cy5.5 (1:20 dilution, clone SK1, BD Biosciences) and anti-CD69 APC (1:50 dilution, clone L78; BD Biosciences) following 16–20 h incubation with stimuli at 37°C, 5% CO_2_. A representative gating strategy for SKW3.HC5 is shown in Supplementary Figure 4, with CD69 mean fluorescence intensity (MFI) values calculated after gating on FSC vs. SSC, single cells, GFP^+^ cells, live cells, CD3^+^CD8^+^ cells and then CD69^+^ cells.

### Statistical Analysis

Statistical significance was determined by non-parametric one-way ANOVA (Kruskal-Wallis test) with *post-hoc* Dunn's multiple comparison test or unpaired Student's *t*-test using Prism 8 (GraphPad) with *p < 0.05, **p < 0.01, and ****p < 0.0001. Error bars indicate the mean ± SEM.

## Results

### Generation of Virus-Specific CD8^+^ T Cell Lines and Clones

**Figure 1:**
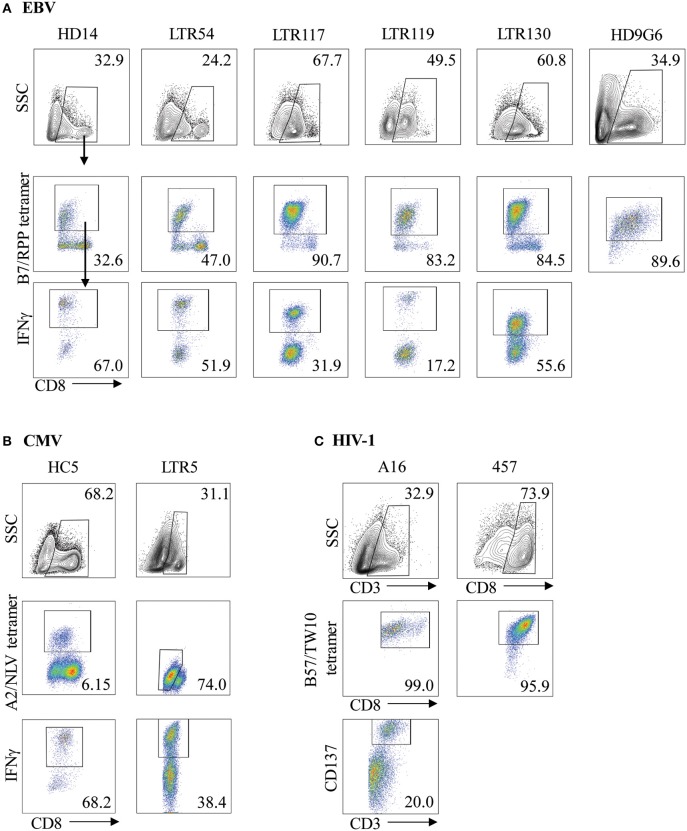
Characterization of virus-specific CD8^+^ T cells. Virus-specific CD8^+^ T cell lines and clones for **(A)** EBV, **(B)** CMV, and **(C)** HIV-1 were examined for specificity following either re-stimulation with HLA-restricted APCs pulsed with cognate viral peptide or bulk PBMC sorting, using both anti-CD8 and specific tetramer. The functionality of virus-specific CD8^+^ tetramer^+^ T cells was assessed either using IFNγ production for T cell lines or via the CD137 activation marker for HIV-1 T cell clones. Cells were gated on FSC vs. SSC, single cells, CD8^+^, CD8^+^tetramer^+^, CD8^+^IFNγ^+^ cells. Representative plots are shown.

Virus-specific CD8^+^ T cells can be generated following stimulation with viral cognate peptide-pulsed autologous PBMCs. To demonstrate both specificity and functionality, *in vitro* expanded T cell lines or clones were co-stained with anti-CD8 and tetramer phenotypic markers for identification of virus-specific T cells. As expected, variations in the frequency of expanded tetramer^+^CD8^+^ T cell lines were observed for both EBV and CMV (Figures 1A,B, middle panels), ranging between 32.6–90.7% (*n* = 6) and 6.15–74.0% (*n* = 2) of the total CD8^+^ T cell population for B7_RPP_ and A2_NLV_, respectively. In addition, CD8^+^ T cell clones raised against the HIV-1 B57_TW10_ epitope in patients A16 and 457 showed very high frequencies following tetramer-specific PBMC bulk sorting (Figure 1C, middle panels), which were similar to the high frequencies observed against the EBV-B7_RPP_ epitope (i.e., HD9G6). To assess the functionality of the EBV- or CMV-specific CD8^+^ T cell lines to produce the pro-inflammatory cytokine IFNγ, cells were restimulated with HLA-restricted APCs pulsed with cognate viral peptide. The frequency of IFNγ production ranged from 17.2 to 67.0% and 38.4 to 68.2% of the CD8^+^ tetramer^+^ T cell population for B7_RPP_ and A2_NLV_, respectively (Figures 1A,B, lower panels). For HD9G6, the functionality of this B7_RPP_-specific CD8^+^ T cell clone is published elsewhere (21). For B57_TW10_-specific CD8^+^ T cell clones A16 and 457, the activation marker CD137, which induces downstream effects of proliferation and cytolytic activity, was used to assess functionality when stimulated with cognate TW10 peptide (Figure 1C), with data for 457 reported elsewhere (20).

### Increased Sensitivity for TCR Cross-Reactivity Detection Using SKW3 Reporter Cells

**Figure 2:**
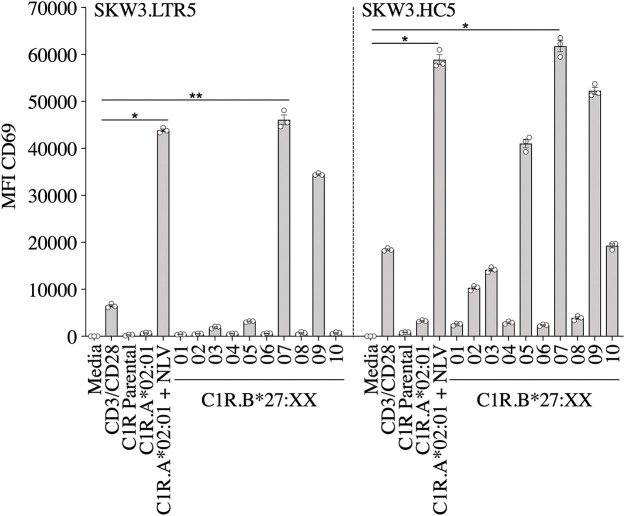
Activation of SKW3.A2_NLV_ TCR cells by HLA-B27–expressing APCs. SKW3.TCR activation was measured using cell surface CD69 upregulation after 16–20 h stimulation with C1R.A*02:01 ± cognate NLV peptide and a panel of C1R.B27 transfectants. CD69 MFI values were calculated after gating on FSC vs. SSC, single cells, GFP^+^ cells, live cells, CD3^+^CD8^+^ cells then CD69^+^ cells. Mean ± SEM are shown (a single experiment with triplicate data is shown from independent biological replicates performed at least twice). Statistical significance denoted by *p < 0.05 and **p < 0. 01 was determined by repeated measures non-parametric ANOVA (Kruskal–Wallis test) with *post-hoc* Dunn's multiple comparison test.

We have previously reported that CMV-specific CD8^+^ T cells raised against A2_NLV_ were differentially cross-reactive toward three HLA-B27 allotypes (B^*^27:07 > B^*^27:09 > B^*^27:05). These T cells were also shown to remain relatively stable following lung transplantation, but increased significantly in response to CMV reactivation (4, 11). Further characterization of the cross-reactive A2_NLV_-specific TCR repertoires from two unrelated individuals showed a striking similarity for the cross-reactive TCR clonotype. Additionally, this study also demonstrated that expression of cross-reactive TCRs in SKW3 cells was a robust system that maintains specificity without the need for continuous *in vitro* expansion of T cell lines or clones for further functional immunoassays (28). In this study, we extended the HLA-B27 allotype panel (B^*^27:01–B^*^27:10) to map the immunogenic profiles of cross-reactive virus-specific T cells (SKW3.LTR5 and SKW3.HC5), which were previously derived from two HLA-A2^+^ donors recognizing both the CMV A2_NLV_ epitope and HLA-B27 molecules (28). Stimulation of both SKW3.LTR5 and SKW3.HC5 with our new panel of C1R.B27 allotypes reconfirmed our previous findings of an immunogenic hierarchy (B^*^27:07 > B^*^27:09 > B^*^27:05) but also revealed additional cross-reactivity toward B^*^27:03 for SKW3.LTR5 and B^*^27:10 > B^*^27:03 > B^*^27:02 for SKW3.HC5. All negative, background (media and C1R Parental, C1R.A^*^02:01) and positive (CD3/CD28 beads, C1R.A^*^02:01+NLV) controls were as expected (Figure 2).

**Figure 3:**
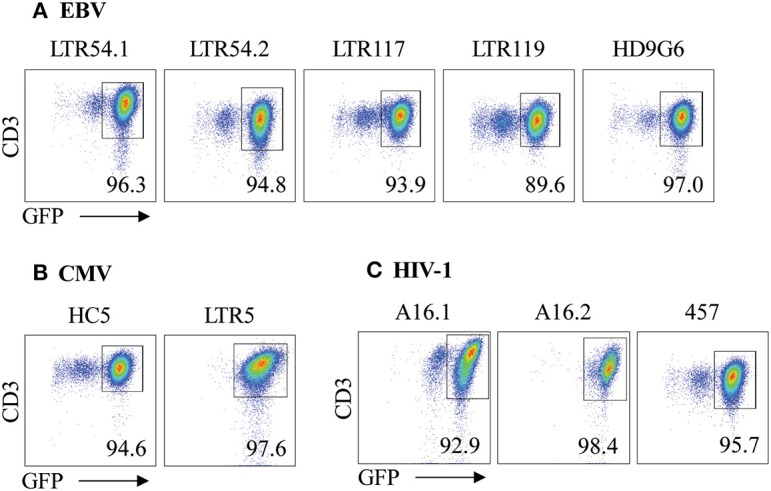
αβTCR expression of SKW3 reporter cells. Retrovirally transduced SKW3 cells expressing cross-reactive virus-specific αβTCRs for **(A)** EBV, **(B)** CMV, and **(C)** HIV-1 were monitored for stable cell surface TCR expression. Cells were gated on FSC vs. SSC, single cells, GFP^+^CD3^+^ cells. Representative plots are shown.

Given the utility of SKW3 reporter cells for profiling TCR cross-reactivity, we adopted this approach to further explore HLA-B27 allorecognition patterns by immunodominant HLA-restricted virus-specific T cells. Here, several cognate peptide-specific CD8^+^ T cells identified using either the ICS immunoassay (i.e., T cell lines) or tetramer sorting (i.e., T cell clones) were sequenced for paired TCR α and β chains. The highest frequency αβTCR was then selected for retrovirus transduction into SKW3 cells (Table 1). Following transduction, extremely high levels of clonality of >90% were easily achieved and maintained by sorting the top 10% of GFP^+^CD3^+^ cells if TCR expression decreased during long-term sub-culturing (Figure 3).

**Table 1:** Virus-specific αβTCR signatures.

**Virus and HLA/epitope**	**TCR**	**α-chain**			**β-chain**			**References**
		**TRAV**	**CDR3**	**TRAJ**	**TRBV**	**CDR3**	**TRBJ**	
EBV	LTR54.1	38-1	CAFSYNNNDMRF	43	4-1	CASSQETGIYTQYF	2-3	
B7_RPP_	LTR54.2	38-1	CAFIQGAQKLVF	54	4-1	CASSQEAFNYEQYF	2-7	
	LTR117	38-1	CAFASSNTGKLIF	43	4-1	CASSQDIWTSGYTF	2-3	
	LTR119	38-2/DV8	CALGGGAQKLVF	54	28	CASRLGLGDREDEKLFF	1-4	
	HD9G6	14/DV4	CAMRDDTGGFKTIF	9	19	CASSISSGVAYEQYF	2-7	(21)
CMV	HC5	3	CAVRGTNARLMF	31	12	CASSSVNEAFF	1-1	(28)
A2_NLV_	LTR5	3	CAVRNNNARLMF	31	12	CASSIVNEAFF	1-1	(28)
HIV-1	A16.1	4	CLVGEVRGGFKTIF	9	4-3	CASSQARGGAETQYF	2-5	
B57_TW10_	A16.2	4	CLVGGEDYKLSF	20	4-3	CASSQARGGAETQYF	2-5	
	457	39	CAVDINTSGTYKYIF	40	10-3	CAISRQGARQETQYF	2-5	(20)

### Dissection of Virus-Specific TCR Cross-Reactivity Toward HLA-B27 Allotypes Reveals Distinct Patterns of Allorecognition

**Figure 4:**
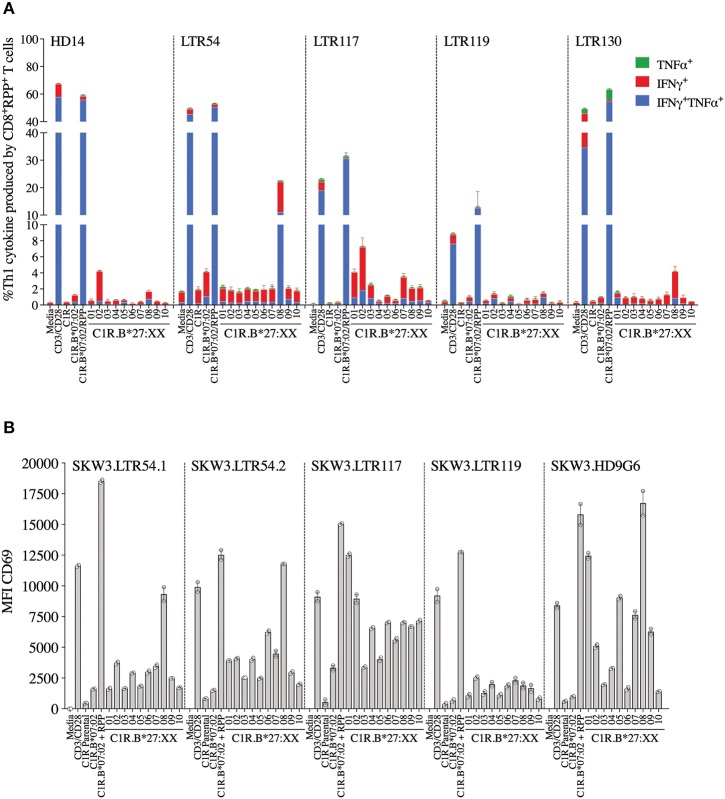
EBV B7_RPP_ allorecognition of HLA-B27 molecules. **(A)** Day 13 *in vitro* expanded B7_RPP_-specific CD8^+^ T cells were stimulated with C1R.B*07:02 ± cognate RPP peptide and a panel of C1R.B27 transfectants before performing a 6 h ICS, with T cell responses measured by the production of Th1 cytokines (i.e., TNFα^+^ or IFNγ^+^ alone or dual TNFα^+^IFNγ^+^) after gating on CD8^+^tetramer^+^ T cells. **(B)** SKW3.TCR activation was measured using cell surface CD69 upregulation after 16–20 h stimulation with C1R.B*07:02 ± cognate RPP peptide and a panel of C1R.B27 transfectants. CD69 MFI values were calculated after gating on FSC vs. SSC, single cells, GFP^+^ cells, live cells, CD3^+^CD8^+^ cells then CD69^+^ cells. Mean ± SEM are shown (single experiments with duplicate data for ICS assay and CD69 assay are shown from independent biological replicates each performed at least twice).

Here we investigated whether EBV-B7_RPP_-specific CD8^+^ T cells could cross-recognize HLA-B27 molecules as a potential trigger of allorecognition. Following validation with the cognate peptide, we re-stimulated our day 13 *in vitro*-expanded B7_RPP_-specific CD8^+^ T cells, which were generated from five EBV-seropositive individuals (HD14, LTR54, LTR117, LTR119, and LTR130), against a panel of HLA-B27-expressing APCs in a 6 h ICS assay with functionality assessed via Th1 cytokine production (i.e., TNFα^+^ or IFNγ^+^ alone or dual TNFα^+^IFNγ^+^). Specificity of the B7_RPP_-specific CD8^+^ T cells was confirmed in all individuals by reactivity to C1R.B^*^07:02 in the presence of cognate RPP peptide. Remarkably, differential patterns of HLA-B27 allorecognition were observed across these individuals. Here, B7_RPP_-specific CD8^+^ T cells from HD14 demonstrated a moderate response to B^*^27:02 and weak responses to B^*^27:08, LTR54 showed a very dominant response to B^*^27:08 only, LTR117 recognized several allotypes with B^*^27:02 > B^*^27:01 > B^*^27:07 and similar levels for B^*^27:03/08/09, LTR119 weakly recognized B^*^27:02/08 and LTR130 moderately responded to B^*^27:08. All negative, background (media and C1R Parental, C1R.B^*^07:02) and positive (CD3/CD28 beads) controls were as expected (Figure 4A, Table 2). Therefore, for the first time, we report a new model of EBV/HLA-B27 cross-reactivity that was observed across multiple individuals.

**Table 2:**
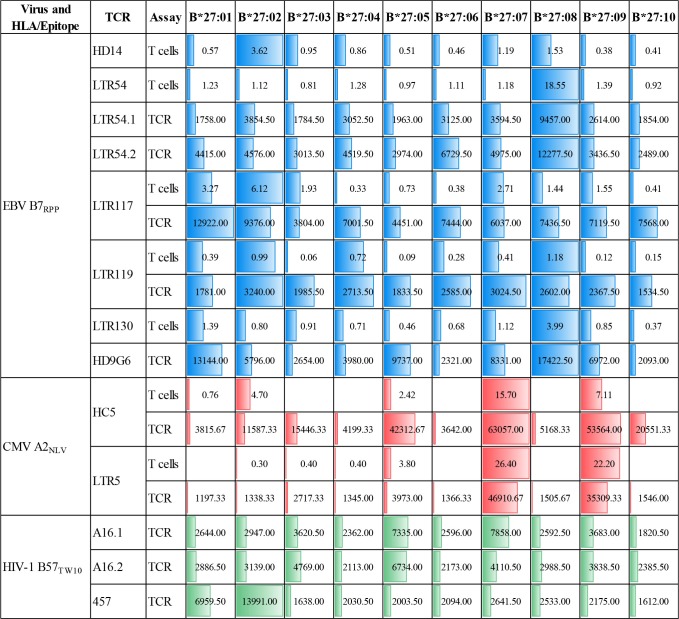
Comparison of T cell cross-reactivity using cellular immunoassays.

*Assays: ICS for T cells, CD69 upregulation for TCR*.

To explore these HLA-B27 allorecognition patterns in greater depth we generated SKW3.TCR cells expressing the B7_RPP_-specific TCR observed at the highest frequency for LTR54 (LTR54.1 and LTR54.2), LTR117 and LTR119, in addition to the published HD9G6 TCR, which showed cross-reactivity toward HLA-B^*^40:01 (21) (Table 1). For LTR54, two TCRs with the same α and β-chain variable regions but different junction regions and CDR3 loops were observed. To investigate whether these contrasting regions were pivotal for allorecognition both TCRs were expressed in SKW3 cells. As observed with the immunogenic hierarchies of CMV A2_NLV_ cross-reactive TCRs for HC5 and LTR5, we demonstrated greater sensitivity of allorecognition using SKW3.TCR reporter cells. The patterns were as follows: LTR54.1 strongly recognized B^*^27:08 and to a weaker extent B^*^27:02/07/04/06/09, whilst in comparison LTR54.2 also strongly responded to B^*^27:08 but weakly recognized alternate B27 allotypes B^*^27:06/01/02/04/07; LTR117 recognized most of the allotypes with the strongest toward B^*^27:01/02 followed by B^*^27:10/08/04/09/06/07; LTR119 showed weak responses across several allotypes with B^*^27:02/07 followed by B^*^27:08/04; and finally HD9G6 strongly recognized B^*^27:08/01 then B^*^27:05/07/09/02/04/03 (Figure 4B, Table 2).

**Figure 5:**
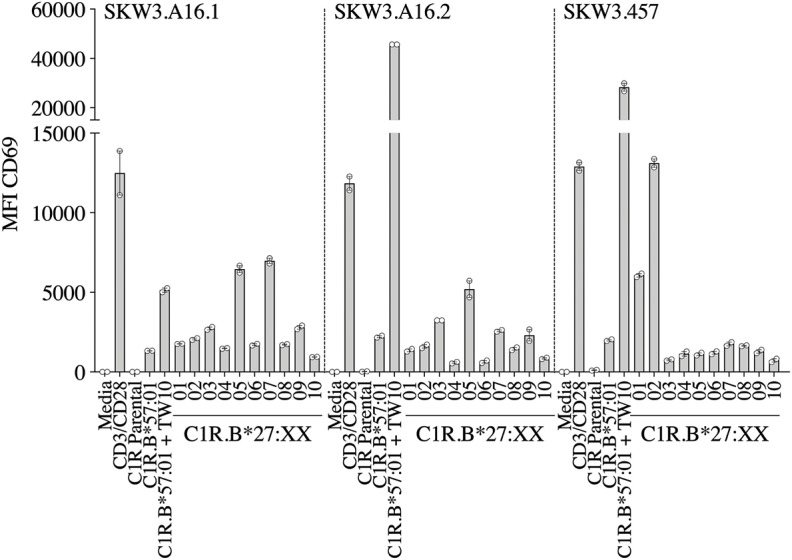
HIV-1 B57_TW10_ allorecognition of HLA-B27 molecules. TCR activation was measured using cell surface CD69 upregulation after 16–20 h stimulation with C1R.B*57:01 ± cognate TW10 peptide and a panel of C1R.B27 transfectants. CD69 MFI values were calculated after gating on FSC vs. SSC, single cells, GFP^+^ cells, live cells, CD3^+^CD8^+^ cells then CD69^+^ cells. Mean ± SEM are shown (a single experiment with duplicate data is shown from independent biological replicates performed at least twice).

A recent report highlighted that HIV-1-specific memory T cells generated from Gag B57_TW10_ epitope can mediate abacavir-induced hypersensitivity reactions through molecular mimicry (20). Therefore, we explored the alloreactive potential of B57_TW10_-specific CD8^+^ T cells toward HLA-B27. TCRs from two T cell clones (A16 and 457) raised against the Gag B57_TW10_ epitope were expressed in SKW3 cells for functional evaluation. Sequencing of the A16 T cell clone revealed two α-chains with different junction regions and CDR3 loops, therefore both TCRs were independently expressed in SKW3 cells (Table 1). Strikingly, comparisons of SKW3.A16.1 and SKW3.A16.2 show a 9-fold difference in recognition of C1R.B^*^57:01 cells presenting cognate TW10 peptide (positive control), suggesting that A16.2 TCR is the primary driver of the cognate peptide recognition. Yet despite this, SKW3.A16.1 recognizes both B^*^27:07 and B^*^27:05 at a similar magnitude to B^*^27:05 allorecognition by SKW3.A16.2. For SKW3.457, responses were biased toward B^*^27:01 and B^*^27:02 (Figure 5, Table 2). Furthermore, we examined whether immunodominant IAV A2_GIL_-specific CD8^+^ T cells, from an alternate RNA virus that induces acute viral infection, could also alloreact toward HLA-B27 allotypes. Here, a total of six healthy donors were screened, and interestingly no significant allorecognition was observed above background levels (Supplementary Figure 5).

### Cognate Viral Peptide Presented by HLA-B27 Allotypes Does Not Confer Additional Immunogenicity

**Figure 6:**
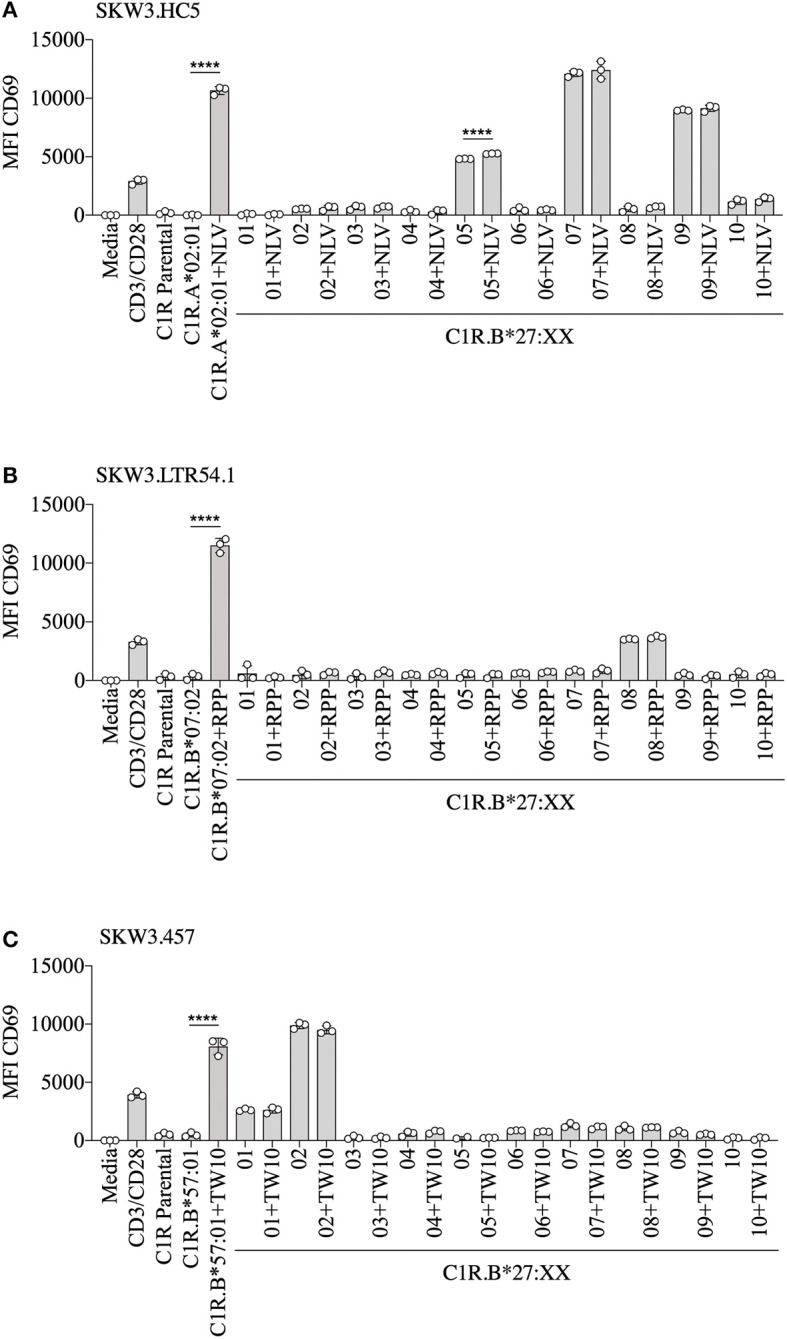
HLA-B27 presentation of cognate viral peptide does not confer additional immunogenicity. **(A)** SKW.HC5 (i.e., CMV A2_NLV_), **(B)** SKW3.LTR54.1 (i.e., EBV B7_RPP_), and **(C)** SKW3.457 (i.e., HIV-1 B57_TW10_) TCR activation was measured using cell surface CD69 upregulation after 16–20 h stimulation with HLA-restricted C1R transfectants ± cognate peptide and a panel of C1R.B27 transfectants. CD69 MFI values were calculated after gating on FSC vs. SSC, single cells, GFP^+^ cells, live cells, CD3^+^CD8^+^ cells then CD69^+^ cells. Mean ± SEM are shown (single experiment with triplicate data). Statistical significance using unpaired Student's *t*-test is denoted by ****p < 0.0001.

To demonstrate that virus-specific TCR cross-reactivity toward HLA-B27 allotypes is *bona fide* and cannot be influenced by the presence of cognate peptide we examined the immune response of SKW.HC5 (i.e., CMV A2_NLV_), SKW3.LTR54.1 (i.e., EBV B7_RPP_), and SKW3.457 (i.e., HIV-1 B57_TW10_) toward a panel of HLA-B27 stimulators in the absence and presence of cognate peptide. All SKW3.TCR lines generated responses to negative, background (media and C1R Parental, C1R.A^*^02:01 or C1R.B^*^07:02 or C1R.B^*^57:01) and positive (CD3/CD28 beads and C1R.A^*^02:01+NLV or C1R.B^*^07:02+RPP or C1R.B^*^57:01+TW10) controls as expected. Importantly, no differences in the magnitude of HLA-B27 allorecognition was observed between APCs in the presence or absence of cognate peptide across all three SKW3.TCR lines. However, surprisingly a statistically significant difference was determined following stimulation of SKW.HC5 with C1R.B^*^27:05+NLV (*p* < 0.0001), which could indicate a role for the presented peptide, however this requires further confirmation (Figure 6).

## Discussion

In this study, we examined the cross-reactive potential of CD8^+^ T cells specific for immunodominant epitopes derived from three different chronic viruses (i.e., CMV, EBV, and HIV-1), presented by commonly expressed HLA (i.e., A2, B7, and B57). We demonstrated that these virus-specific CD8^+^ TCRs were capable of vigorous cross-reactivity toward specific HLA-B27 allotypes, and that the immune responses were hierarchical and varied considerably across the three chronic viruses.

Whilst, we previously reported a defined pattern of strong HLA-B27 T cell cross-reactivity (B^*^27:07 > 09 > 05) by CMV A2_NLV_ CD8^+^ TCRs for both LTR5 and HC5 (11, 28), this study extended the number of B27 subtypes examined and revealed additional cross-reactivity toward B^*^27:10 > 03 > 02 for HC5. Interestingly, despite subtle sequence differences in the CDR3 regions of both the α- and β-chains (2 and 1 amino acids, respectively) between LTR5 and HC5, the fine specificity of strong TCR interactions with B^*^27:07/09 allotypes were maintained. The data suggests that the composition of the allopeptide(s) presented by each HLA-B27 allomorph are similar or alternatively, of high affinity and that molecular flexibility of the CDR3 loops aids promotion of TCR engagement (33, 34). In contrast, weaker responses toward B^*^27:02/03/10 show delineation in TCR interaction, with LTR5 not demonstrating recognition of these allotypes, which may be due to weak TCR interactions below the assay sensitivity threshold. This suggests that the allopeptide contribution required to form the ternary complex is impacted by the variability observed in the CDR3 regions, which is supported by structural studies of the murine 2C TCR demonstrating that variations in the CDR3α loop dictated TCR affinity and cross-reactivity between distinct ligands (35). Indeed, the importance of the TCR variable domains in promoting high affinity interactions with pHLA complexes was also shown with the human HLA-A2-restricted cancer antigen MART-1 (36). Further investigations are required to decipher the allopeptide(s) presented by these HLA-B27 allotypes and determine their exact role in conferring cross-reactivity.

We next examined the magnitude of cross-reactivity exhibited by EBV-specific B7_RPP_ CD8^+^ T cells toward HLA-B27 allotypes. In the five HLA-B7^+^ individuals, including a healthy donor and immunosuppressed patients, allorecognition resulted in production of proinflammatory Th1 cytokines (IFNγ and TNFα) mainly toward either B^*^27:02 or B^*^27:08. Although, it should be noted that an additional screen of four healthy donors showed no HLA-B27 cross-reactivity, suggesting that allorecognition is driven by private TCR usage. The B7_RPP_ CD8^+^ TCR repertoires were sequenced for three of these individuals to determine their clonotypic profiles. Interestingly, only two clonotypes were observed for LTR54 (i.e., LTR54.1 and LTR54.2), which differed in the CDR3 and J regions of both TCRα- and β-chains. Both TCRs were expressed in SKW3 cells for further functional validation. Additionally, comparison of the B7_RPP_ CD8^+^ TCR clonotypes showed a high degree of similarity between LTR54.1 and LTR117, with differences only noted in the CDR3α- and β-loops. Whilst, LTR119 and the previously reported B7_RPP_ CD8^+^ T cell clone, HD9G6 (21), are vastly different from the other TCRs in this cohort. Interestingly, the strongest TCR cross-reactivity was relatively restricted to B^*^27:08 (LTR54.1, LTR54.2, HD9G6) and B^*^27:02 (LTR117), although there was a degree of allorecognition toward other subtypes for most TCRs. These observations highlight that both private (i.e., LTR119 and HD9G6) and shared (i.e., LTR54.1 and LTR117) TCR specificities contribute to cross-reactivity, and that the cross-reactive pattern diversity is dependent on the Vβ region (2, 15, 16). Furthermore, Amir et al. (2) also reported that T cell clones with identical Vβ regions from the same individual held private specificities and generated different alloreactions. For example, in donor BDV a T cell clone raised against CMV B7_RPHERNGFTVL_ with TCR Vβ7.2 recognized DRB1^*^08:01, whilst another T cell clone from same individual with the identical Vβ did not. Additionally, in donor FKR an influenza A2_GIL_ T cell clone with Vβ17 recognized allogeneic HLA-B^*^64:01 but another T cell clone with the identical Vβ failed. These T cell clones had private differences in TCR sequence, which effectively abrogated alloreactivity.

For the herpesvirus TCRs, the allorecognition hierarchy remained relatively static for the strongest responses, but this was not observed in the case of HIV-1 B57_TW10_ CD8^+^ TCRs in that 457 and A16 TCRs were completely focused toward different HLA-B27 allotypes. Here, we show that 457 TCR cross-reacted strongly toward B^*^27:02 > 01, with two TCRs derived from A16 strongly recognizing B^*^27:05/07 for A16.1 and B^*^27:05 for A16.2. Comparison of their TCRs revealed that their signatures were completely different, supporting that the B57_TW10_ specificity is driven by private TCR usage in these two individuals. Particularly of interest was the dual expression of two different TCRα-chains from the A16 T cell clone, which when independently expressed in SKW3 reporter cells, showed reactivity differences not only toward the B27 subtypes but also importantly against the cognate antigen. We observed that the A16.2 TCR was geared toward cognate antigen recognition, with the A16.1 TCR being more alloreactive. Up to 30% of human peripheral T cells naturally express dual TCRα-chains (37), with multiple studies demonstrating that the allelic inclusion facilitates a heightened immune response by providing an additional chance for antigen recognition and engagement [extensively reviewed in (38)].

**Figure 7:**
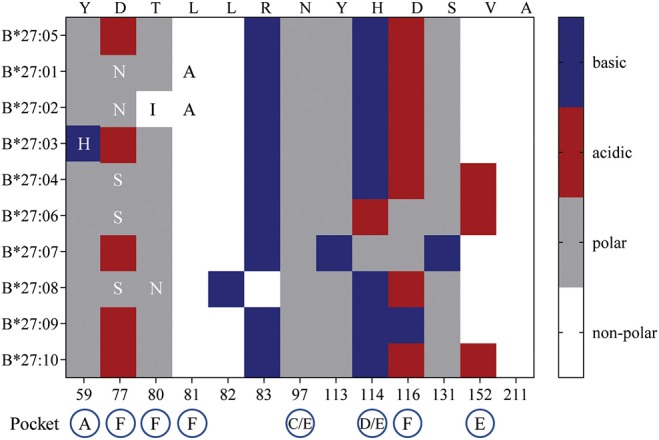
HLA-B27 polymorphisms and amino acid chemical properties. B27:01-B*27:04, B*27:06-B*27:10 amino acid polymorphisms were compared to the consensus sequence for B*27:05. The chemical properties of substituted residues and their influence on HLA-I peptide-binding pockets are detailed.

So, what drives the preferential HLA-B27 allorecognition displayed by these virus-specific TCRs? Undoubtedly, the polymorphic nature of the B27 molecule itself greatly influences the peptide cargo being displayed to surveying T cells (Figure 7). In our study, the A2_NLV_ CD8^+^ TCRs preferentially bind to B^*^27:07/09/05, which differ by 1 (B^*^27:09) and 5 (B^*^27:07) amino acids compared to the consensus B^*^27:05 allotype. These polymorphisms directly impact the D/E (position 114, peptide contacts P5–P7) and F (position 116, peptide contact P9) peptide-binding pockets, which are known immunological hot spots for non-permissive HLA mismatches in transplantation (39–43). Given that the public A2_NLV_ CD8^+^ TCR co-recognizes these three molecules and their relative impact on the peptide-binding pockets D/E and F, suggests that each may be presenting an alternate allopeptide with affinity above a threshold to promote TCR engagement. This is supported by the prototypic HLA-B8-restricted LC13 TCR which is capable of engaging with HLA-B^*^44:05 presenting either an allotype or mimotope (18). In addition, we cannot exclude that the same allopeptide may also be presented by all HLA-B27 molecules, with allorecognition being impacted by differences in conformational flexibility. Indeed, a study by Loll et al. demonstrated that a HLA-B27-derived self-peptide derived from vasoactive intestinal peptide receptor type 1 (epitope; RRKWRRWHL) is differentially presented by AS-associated B^*^27:04 and B^*^27:05 compared to the non-AS-associated B^*^27:06 and B^*^27:09 due to structural variations in molecular dynamics (44). For B7_RPP_ CD8^+^ TCRs, recognition was focused toward B^*^27:08/02/01, with B^*^27:01 and B^*^27:02 differing by a single amino acid (position 80) and both differing from B^*^27:08 by 5 amino acids (positions 77, 80–83), all of which also influence the F peptide-binding pocket (Figure 7). Finally, for the B57_TW10_ CD8^+^ TCRs we observed completely divergent recognition of B27 allotypes by 457 (B^*^27:01/02) and A16 (B^*^27:05/07). However, a common feature is involvement of the F pocket at positions 80 and 116, respectively. Interestingly, the F pocket not only determines the carboxy terminal motif of HLA-I peptides (45), but in other HLA-B27 allotypes has also been shown to affect anchoring sites (i.e., B^*^27:06; P3, PΩ-2, and PΩ) (46). Moreover, positions 114 and 116 are important for the chaperone tapasin, involved in loading of optimal peptides on HLA-I molecules (47, 48). Whilst, the identification of allopeptides has been a major limiting factor hampering translational impact in clinical studies, further investigations are warranted to assess the true impact of T cell cross-reactivity.

Here we analyzed three HLA-B27 cross-reactivity models, including our newly identified EBV/HLA-B27 model, using T cell lines/clones and the more fine-tuned TCR-specific SKW3 cell lines, to reveal the diversity and breadth of cross-reactivity against different HLA-B27 allotypes. Specifically, we showed that cross-reactive TCRs (LTR5, HC5, LTR119, A16.1) derived from the three heterologous viruses were capable of recognizing B^*^27:07, with cross-reactive TCRs from two viruses recognizing either B^*^27:01 (LTR117, HD9G6, 457), B^*^27:02 (LTR119, 457), or B^*^27:05 (HC5, A16.1, A16.2). Collectively, this study demonstrated selective TCR cross-reactivity toward HLA-B27 allotypes by chronic latent viruses, which may evoke clinically relevant alloreactivity following transplantation.

## Data Availability Statement

The raw data supporting the conclusions of this article will be made available by the authors, without undue reservation, to any qualified researcher.

## Ethics Statement

All study participants provided written consent, with ethics approval granted by The Alfred Hospital [Victoria, Australia; ethics no. 175/02, lung transplant recipient (LTR)5, LTR54, LTR117, LTR119, LTR130], Monash University (Victoria, Australia; ethics no. 10950, healthy control/donor HC5, HD14), Australian Bone Marrow Donor Registry (NSW, Australia; ethics no. 2012/05, healthy donors NM003, NM008, NM009,NM010, NM014, NM016), Royal Perth Hospital (Western Australia, Australia; ethics no. HREC 1999-021, HIV-1^+^ patients A16, 457) and Leiden University Medical Center (Leiden, The Netherlands; buffy coat donation, HD9G6) in accordance with the Declaration of Helsinki. The patients/participants provided their written informed consent to participate in this study.

## Author Contributions

LR, HH, LD'O, and NM contributed conception and designed of the study. LR, HH, JS, LD'O, TN, and NM performed the experiments. LR, HH, LD'O, TN, and NM analyzed the data. FC, JR, TK, AP, and NM made significant contributions to reagents. LR and NM wrote the manuscript. All authors read and approved the manuscript.

### Conflict of Interest

The authors declare that the research was conducted in the absence of any commercial or financial relationships that could be construed as a potential conflict of interest.

## Supplementary Material

Supplementary Figure 1 HLA cell surface expression of APCs. APCs were stained with primary antibody either **(A)** pan-HLA-I W6/32 or **(B)** anti-HLA-B7/27 ME1, followed by secondary goat anti-mouse IgG PE. A secondary (2°) antibody alone control was used for background staining. MFI was calculated after gating FSC vs. SSC then primary antibody histogram. Representative plots are shown.

Supplementary Figure 2 Gating strategy for specificity and functionality of virus-specific CD8^+^ T cells. Representative virus-specific CD8^+^ tetramer^+^ T cells was assessed for either **(A)** IFNγ production with cells gated on FSC vs. SSC, single cells, live cells, CD8^+^, CD8^+^tetramer^+^, and CD8^+^IFNγ^+^ cells or **(B)** CD137 activation with cells gated on FSC vs. SSC, CD3^+^ or CD8^+^, CD8^+^tetramer^+^, and CD3^+^CD137^+^ cells.

Supplementary Figure 3 Gating strategy for SKW3.TCR expression. Representative cell surface TCR expression for SKW3.LTR119 is shown. Cells were gated on FSC vs. SSC, single cells, GFP^+^CD3^+^ cells.

Supplementary Figure 4 Gating strategy for CD69 upregulation assay. Representative CD69 cell surface upregulation for SKW3.HC5 is shown following stimulation with media, C1R.A^*^02:01+NLV (cognate peptide), C1R.B^*^27:01 (non-cross-reactive B27 allele) and C1R. B^*^27:07 (cross-reactive B27 allele). CD69 MFI values were calculated after gating on FSC vs. SSC, single cells, GFP^+^ cells, live cells, CD3^+^CD8^+^ cells, and then CD69^+^ cells.

Supplementary Figure 5 IAV A2_GIL_ allorecognition of HLA-B27 molecules. **(A)** Representative gating strategy of NM003 d13 A2_GIL_-specific CD8^+^ T cells stimulated with C1R.A^*^02:01+GIL peptide; FSC vs. SSC, single cells, live cells, CD8^+^, CD8^+^tetramer^+^ and IFNγ^+^TNFα^+^ cells. **(B)** Day 13 *in vitro* expanded A2_GIL_-specific CD8^+^ T cells were stimulated with C1R.A^*^02:01 ± cognate GIL peptide and a panel of C1R.B27 transfectants before performing a 6 h ICS, with T cell responses measured by the production of Th1 cytokines (i.e., TNFα^+^ or IFNγ^+^ alone or dual TNFα^+^IFNγ^+^) after gating on CD8^+^tetramer^+^ T cells. Mean ± SEM are shown (single experiment with duplicate data).

Supplementary Table 1 HLA class I typing of study participants.

## Abbreviations

HD: healthy donor
HLA: human leukocyte antigen
LTR: lung transplant recipient
PBMC: peripheral blood mononuclear cells
pHLA: peptide-HLA
TCR: T cell receptor.
