# Supplementary figures and images for: Preferential HLA-B27 Allorecognition Displayed by Multiple Cross-Reactive Antiviral CD8+ T Cell Receptors
Source: Front Immunol. 2020 Feb 19;11:248. doi: 10.3389/fimmu.2020.00248 (PMC7042382; doi:10.3389/fimmu.2020.00248)

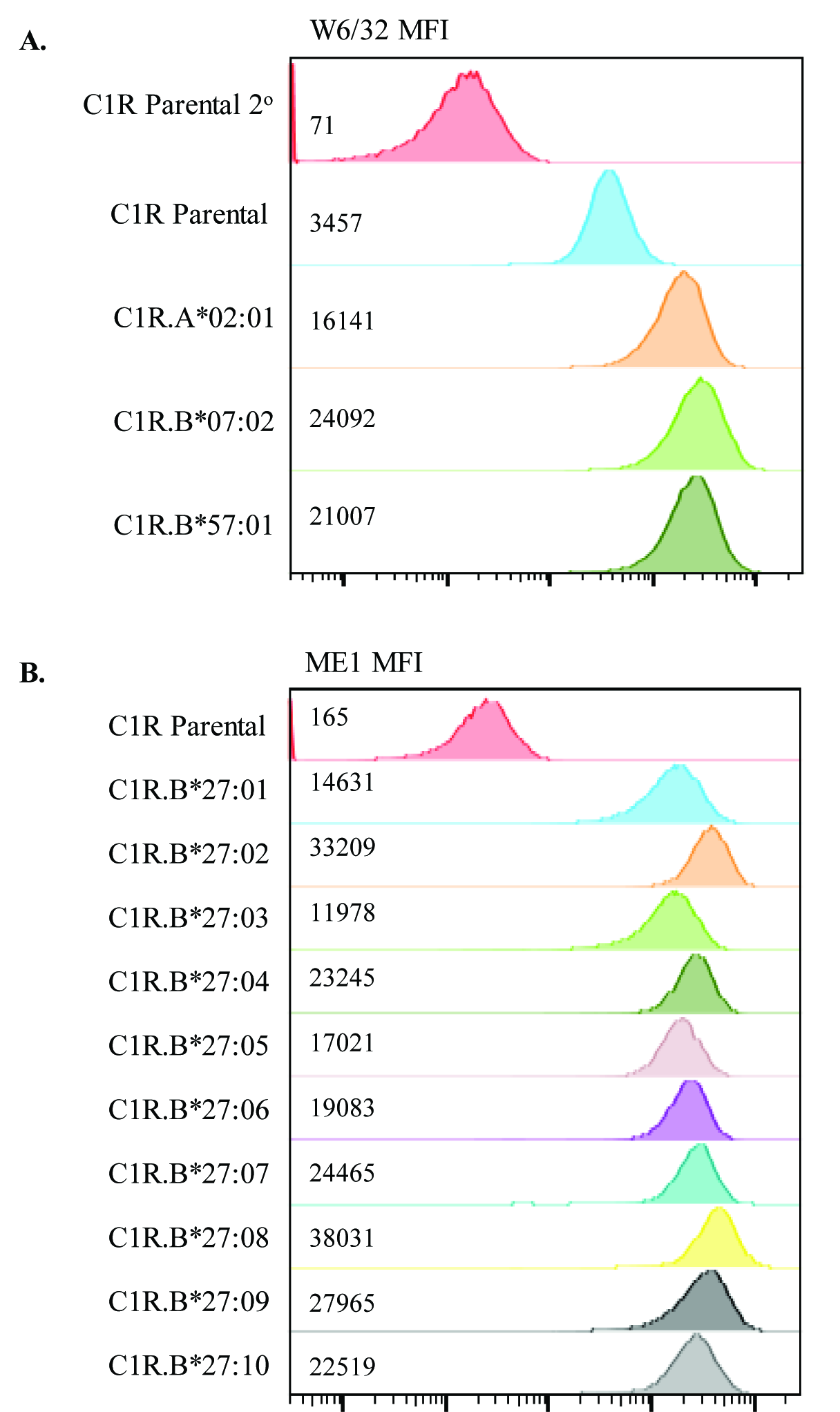

Supplement: Supplementary Figure 1 — HLA cell surface expression of APCs. APCs were stained with primary antibody either (A) pan-HLA-I W6/32 or (B) anti-HLA-B7/27 ME1, followed by secondary goat anti-mouse IgG PE. A secondary (2°) antibody alone control was used for background staining. MFI was calculated after gating FSC vs. SSC then primary antibody histogram. Representative plots are shown. [file Image_1.tif]

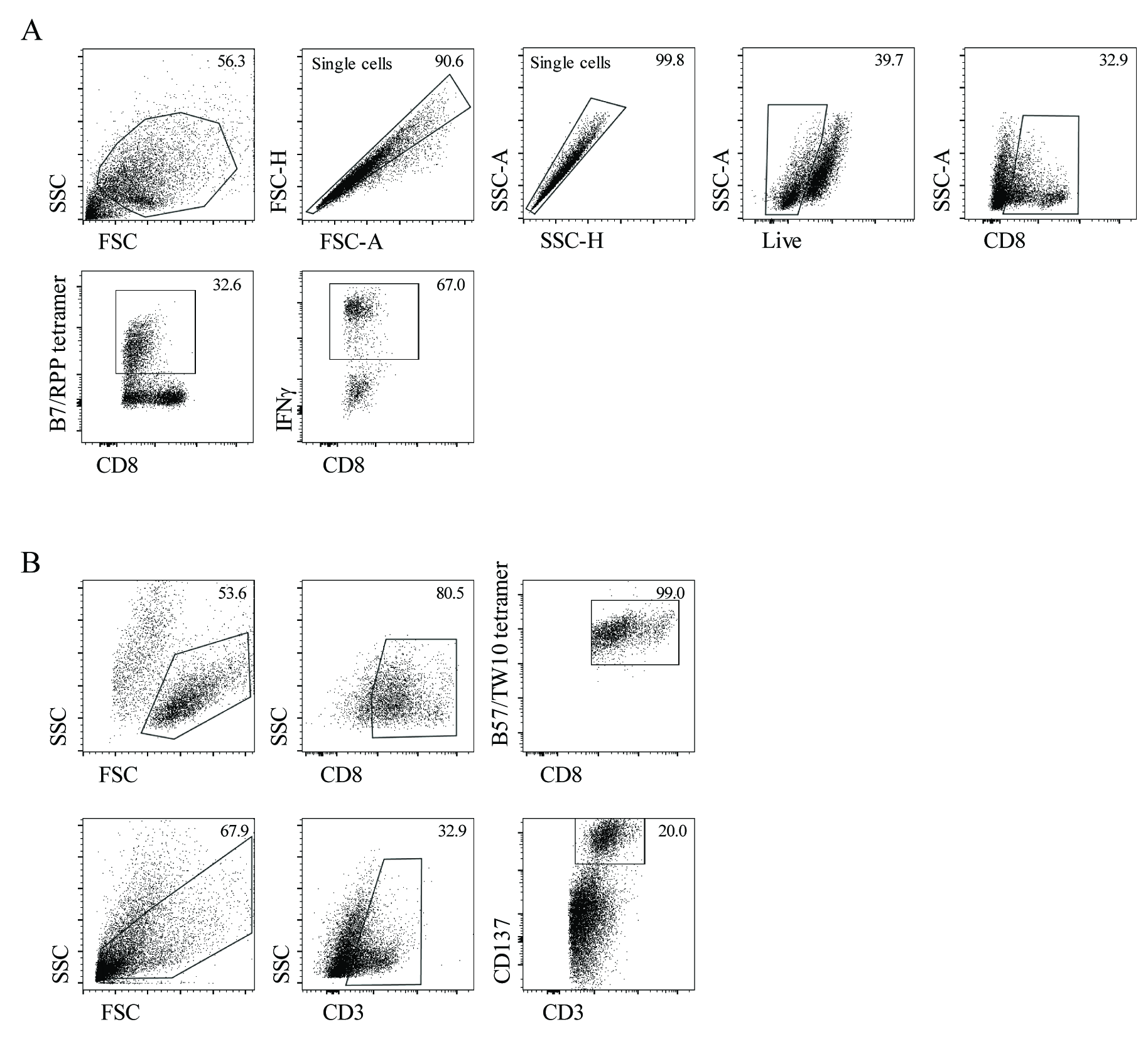

Supplement: Supplementary Figure 2 — Gating strategy for specificity and functionality of virus-specific CD8+ T cells. Representative virus-specific CD8+ tetramer+ T cells was assessed for either (A) IFNγ production with cells gated on FSC vs. SSC, single cells, live cells, CD8+, CD8+tetramer+, and CD8+IFNγ+ cells or (B) CD137 activation with cells gated on FSC vs. SSC, CD3+ or CD8+, CD8+tetramer+, and CD3+CD137+ cells. [file Image_2.tif]

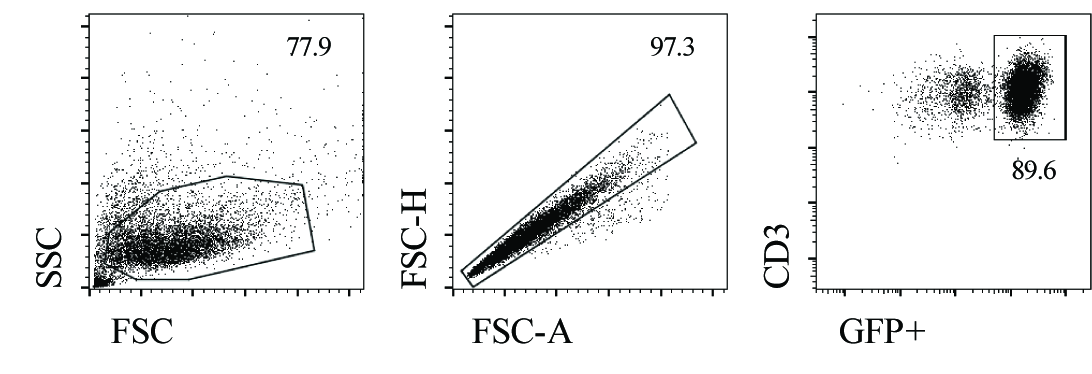

Supplement: Supplementary Figure 3 — Gating strategy for SKW3.TCR expression. Representative cell surface TCR expression for SKW3.LTR119 is shown. Cells were gated on FSC vs. SSC, single cells, GFP+CD3+ cells. [file Image_3.tif]

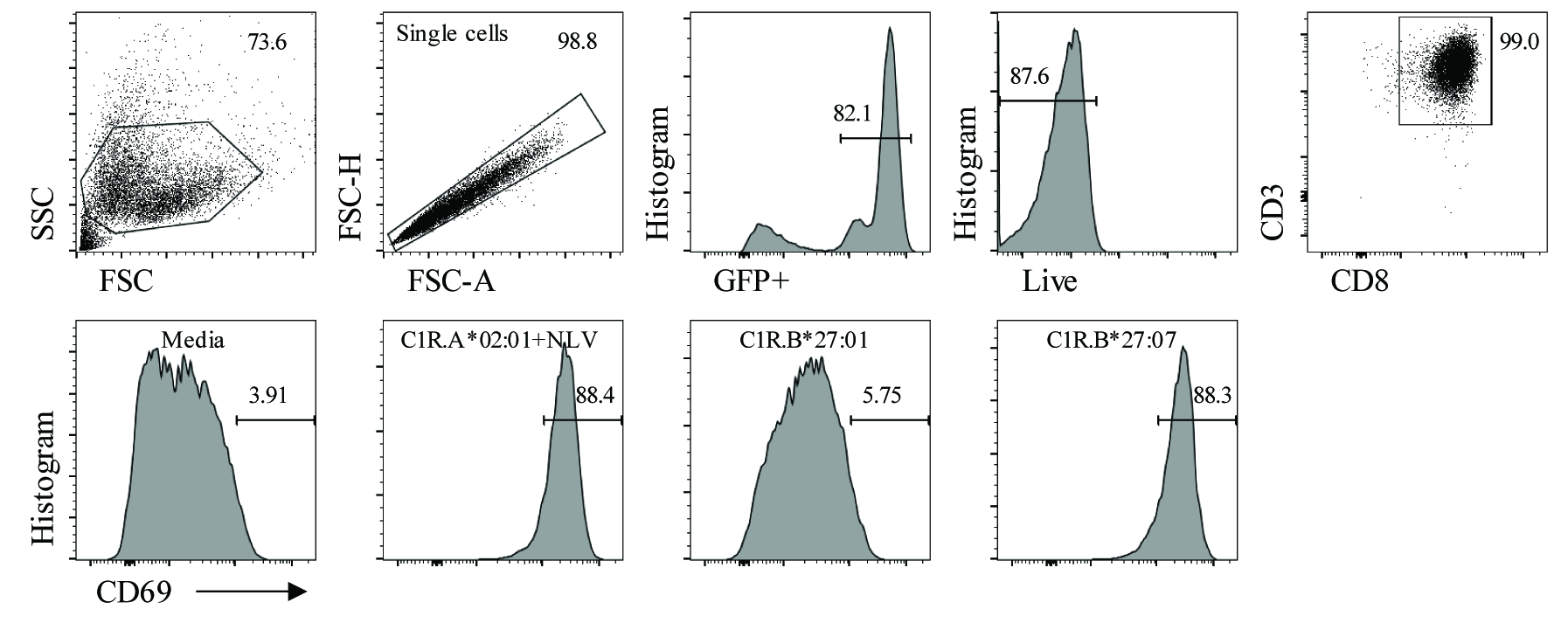

Supplement: Supplementary Figure 4 — Gating strategy for CD69 upregulation assay. Representative CD69 cell surface upregulation for SKW3.HC5 is shown following stimulation with media, C1R.A*02:01+NLV (cognate peptide), C1R.B*27:01 (non-cross-reactive B27 allele) and C1R. B*27:07 (cross-reactive B27 allele). CD69 MFI values were calculated after gating on FSC vs. SSC, single cells, GFP+ cells, live cells, CD3+CD8+ cells, and then CD69+ cells. [file Image_4.tif]

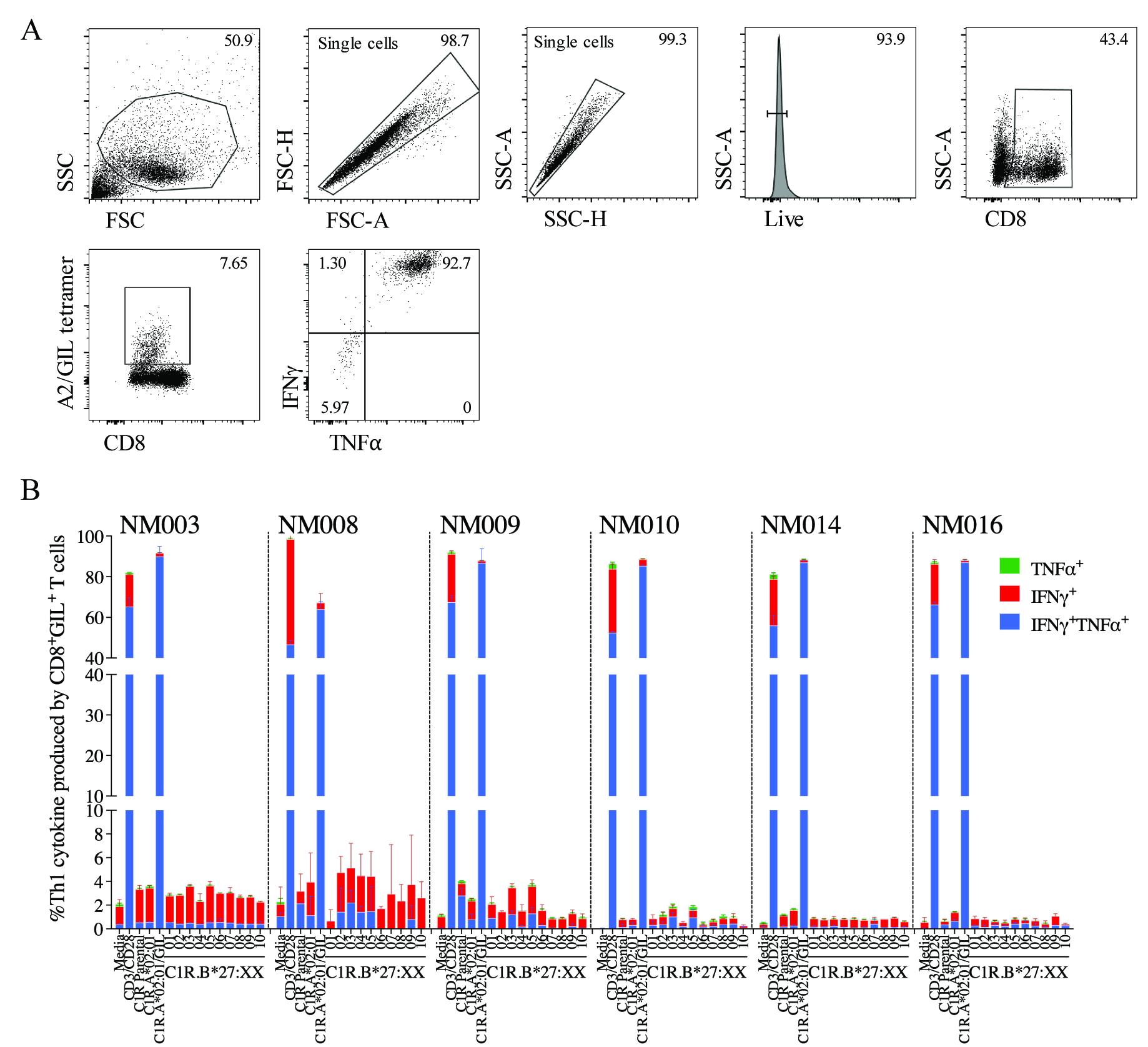

Supplement: Supplementary Figure 5 — IAV A2GIL allorecognition of HLA-B27 molecules. (A) Representative gating strategy of NM003 d13 A2GIL-specific CD8+ T cells stimulated with C1R.A*02:01+GIL peptide; FSC vs. SSC, single cells, live cells, CD8+, CD8+tetramer+ and IFNγ+TNFα+ cells. (B) Day 13 in vitro expanded A2GIL-specific CD8+ T cells were stimulated with C1R.A*02:01 ± cognate GIL peptide and a panel of C1R.B27 transfectants before performing a 6 h ICS, with T cell responses measured by the production of Th1 cytokines (i.e., TNFα+ or IFNγ+ alone or dual TNFα+IFNγ+) after gating on CD8+tetramer+ T cells. Mean ± SEM are shown (single experiment with duplicate data). [file Image_5.tif]
