# Supplementary material for: Preferential HLA-B27 Allorecognition Displayed by Multiple Cross-Reactive Antiviral CD8+ T Cell Receptors
Source: Front Immunol. 2020 Feb 19;11:248. doi: 10.3389/fimmu.2020.00248 (PMC7042382; doi:10.3389/fimmu.2020.00248)
Supplement: Supplementary Table 1 — HLA class I typing of study participants. [file Table_1.DOCX]

**Supplementary Table 1. HLA class I typing of study participants.**

|  | HLA class I | | |
| --- | --- | --- | --- |
| Participant | A | B | C |
| LTR5 | 02, 03 | 07, 18 | 07 |
| LTR54 | 02, 03 | 07 | 07 |
| LTR117 | 01, 03 | 07, 08 | 07 |
| LTR119 | 01, 24 | 07, 18 | 07, 12 |
| LTR130 | 02, 03 | 07 | 05, 07 |
| HC5 | 02:01, 11 | 51, 61 | 02, 07 |
| HD14 | 02:01, 03:01 | 07:02, 45:01 | 06:02, 07:02 |
| NM003 | 01, 02 | 07, 08 |  |
| NM008 | 01, 02 | 07, 08 |  |
| NM009 | 01:01, 03:01 | 07:02, 08:01 |  |
| NM010 | 01, 02 | 07, 08:01 |  |
| NM014 | 01, 02 | 07, 08 |  |
| NM016 | 01:01, 02:01 | 07:02, 08:01 |  |
| A16 | 03:01, 32:01 | 14:01, 57:01 | 06:02, 08:02 |
| 457 | 01:01, 03:01 | 08:01, 57:01 | 06:02, 07:01 |
| HD9G6 | 02, 03 | 07, 35 | 04, 07 |
